# Promoting physical activity interventions in communities with poor health and socio-economic profiles: A process evaluation of the implementation of a new walking group scheme

**DOI:** 10.1016/j.socscimed.2016.09.035

**Published:** 2016-11

**Authors:** Sarah Hanson, Jane Cross, Andy Jones

**Affiliations:** a Norwich Medical School, University of East Anglia, Norwich, Norfolk, NR4 7TJ, UK; b School of Health Sciences, University of East Anglia, Norwich, Norfolk, NR4 7TJ, UK

**Keywords:** Walking groups, Physical activity, Public health, Health equity, Process evaluation

## Abstract

Walking groups have known health benefits but may not operate in communities with the greatest health needs, leading to the potential for increasing health inequity. This study examined the process of implementing a new volunteer led walking group scheme in a deprived community in England with poor physical activity, health and socio-economic indicators. Documentary evidence and semi-structured interviews with stakeholders and volunteer walk leaders undertaken at the beginning and end of the funding period were analysed thematically. It was found that utilising community-based assets, forming collaborative partnerships with health and non-health organisations and ongoing sustainability issues were all factors that affected the scheme's effective implementation. Passive recruitment methods and mass publicity did not attract participants who were representative of their community. The findings firstly suggest the necessity of identifying and mobilising community based assets at the ‘grass roots’ in deprived communities during the preparatory stage to access those in greatest need and to plan and build capacity. Secondly, the findings highlight the key role that health professionals have in referring those in poorest health and the inactive into walking interventions. In the new era of fiscally constrained public health embedded within local authorities these findings are pertinent in supporting the utilisation of local assets to address entrenched physical inactivity and inequity within deprived communities.

## Highlights

• Factors in walking group implementation revealed in two phase process evaluation.
• Volunteer Walking Champions were not representative of more deprived communities.
• Health practitioner referrals seen as a ‘missing link’ to reach those with most need.
• Implementation was limited by not utilising or mobilising community based assets.
• Short term funding affected long-term capacity building with partners.

## 1 Introduction

Physical activity has wide-ranging long-term health benefits and reduces the risk of chronic disease (Friedenreich et al., 2010, Reiner et al., 2013). Even small increases in activity could benefit population health, with the largest gains coming from inactive individuals becoming moderately active doing 20 min of brisk walking each day (Ekelund et al., 2015). The simplicity of walking, associated with little cost, makes it economically accessible and thus one of the best ways to achieve recommended daily amounts of physical activity (ACSM, 2011). However, in England it has been estimated that 8% of the population do not walk continuously for five minutes in a four week period (Farrell et al., 2013).

Walking can be promoted through outdoor health walks in community settings (Public Health England, 2014). Walking groups have been shown to confer multiple physiological and psychological health benefits with good adherence and few side effects and are potentially a useful intervention for those who would benefit from increasing physical activity (Hanson and Jones, 2015a).

Physical activity interventions can be effective in low income groups but have the potential to increase intervention-generated inequalities (Bull et al., 2014). Preventative interventions are known to be socially patterned and more likely to be successful amongst the more affluent, a process which has been termed as the ‘inverse prevention law’ (Acheson, 1998). It has therefore been cautioned that all processes in the planning and delivery of health promoting interventions have the potential to widen inequity between groups, the implications of which are important to researchers, practitioners and policy makers (White et al., 2009).

As with other health promoting interventions there are therefore health inequity concerns. Firstly, without effective targeting of areas with the greatest health and socio-economic need, walking groups might not be set up in communities that need them most (Hanson and Jones, 2015b). Secondly, walking interventions tend to be taken up by white, well-educated, middle aged women (Foster et al., 2011). Finally, recent research with a walking group operating in an area of health and socio-economic deprivation found barriers for those very people for whom walking groups could potentially offer the greatest benefit (Hanson et al., 2016). For example, walking groups were viewed by participants as being of little purpose with a poor understanding of the health benefits of walking per se. Further, the group format itself represented a barrier by creating a general apprehension about what to wear, the fitness levels needed and an expectation of socialising with others in the group (Hanson et al., 2016). Walking groups could be well placed to promote the physical activity needs of those with intellectual disabilities as walking is a preferred form of physical activity (Finlayson et al., 2009). People with intellectual disability experience significant health inequalities and lead more sedentary lifestyles than the general population, they are also under-investigated and the best ways of supporting a more physically active, and less sedentary, lifestyle is a health improvement priority (Hanson and Jones, 2015a, Melville et al., 2015, Mitchell et al., 2013).

Setting up and promoting walking groups in deprived communities for individuals whose health would benefit the most therefore poses clear challenges. Unless addressed, there is the potential for walking groups to widen preventable health inequity.

‘Walk Norwich’ is a community wide intervention in the city of Norwich, England. It is part of the ‘Walking Cities’ project funded by the Department of Health (DH) in 2014 implementing walking initiatives to encourage local journeys on foot (Department for Transport, 2013). The new funding enabled Norwich City Council to develop different walking programmes, involving school children, lift-share plans (car-pooling) for people in work, plus a walking group initiative with short group walks for the inactive led by volunteer ‘Walking Champions’ (Norwich City Council, 2015a, Norwich City Council, 2015b).

The Walking Champion initiative in deprived communities in Norwich offered an opportunity for evaluation using natural experiment principles (Craig et al., 2012). The initiative was not under the control of the researchers and this enabled evaluation under ‘real world’ circumstances. The recent Cochrane review (Baker et al., 2015) suggested that process evaluations should be undertaken as they provide valuable information on potential barriers and facilitators plus an indication of how successfully an intervention has been implemented. Process evaluation focuses on the processes used throughout the intervention and aims to understand what went well and what went wrong. It does this by examining implementation; the mechanisms through which the intervention produces results and contextual factors external to the intervention which may influence its implementation (Moore et al., 2015).

This paper presents a process evaluation of a new walking group initiative within a community in England with poor physical activity, health and socio-economic indicators. Data were collected from semi-structured interviews with stakeholders responsible for the design, implementation and sustainability of the scheme and volunteer Walking Champions, the name given to the volunteers who led the group walks. Our aims were to identify the essential elements that stakeholders perceived as facilitating or presenting barriers to the implementation, impact and sustainability of the scheme and to produce a set of recommendations for how to best implement physical activity interventions in deprived communities to maximise their impact.

The study was given a favourable ethical opinion by the ethics committee of the Faculty of Medicine and Health Sciences at the University of East Anglia in July 2014.

## 2 Methods

This qualitative study was organised around the key functions of a process evaluation. The description of the intervention and its logic; how the delivery was implemented; the mechanisms through which the intervention produced results; contextual factors external to the intervention which may influence implementation and anticipated outcomes (Moore et al., 2015).

### 2.1 Setting of the walking programme

The group walking scheme was a programme of short health walks (of approximately one mile) in areas of multiple deprivation in Norwich and, where possible, connected to a cycleway (Department for Transport, 2013). The walks were mapped and risk-assessed by an experienced walks co-ordinator with responsibility for day-to-day management of the scheme when it was first set up. The walks ran approximately 3–4 times during the week. They were promoted to the public with brochures and posters in libraries, some doctors' surgeries and community centres. In the event, usually 2–4 people attended except when the walks were run in partnership with an organisation for adults with learning disabilities when 6–8 attended with an assistant. The area is urban with high density housing but with access to city parks, footpaths and riverside walkways, which were utilised for the group walks, led by the Walking Champions. The main focus was the Heartsease area with Bowthorpe and Mile Cross as examples of other neighbourhoods. All targeted areas had deprivation scores worse than the English average. For example, Heartsease is amongst the 40% most deprived and Bowthorpe and Mile Cross amongst the 20% most deprived neighbourhoods in England, based on the 2015 Indices of Multiple deprivation (Department for Communities and Local Government, 2015). Only 29% of people in Norwich are estimated to meet government guidelines of 150 min of moderate activity per week (Sport England, 2013).

### 2.2 Participants and interview process

A previous study examined the barriers and enablers for walking group participants (Hanson et al., 2016). Therefore the focus of this study was the process of implementing a walking scheme from the point of view of those organising it. Our participants were two groups of people, stakeholders responsible for setting up and managing the scheme and volunteer Walking Champions who led the walks. The first were key stakeholders suggested by the scheme's organisers. These stakeholders were involved in the planning, bid writing and implementation of the scheme and included people involved in the day-to-day management; from the public health department; the local clinical commissioning group; DH (the funding source) and a Councillor from Norwich City Council. All stakeholders were invited and agreed to participate. In total there were 12 participants, six men and six women. Two participants did not participate in the follow-up interview and a further informant was only suggested at the second time point.

The second group of participants were volunteer Walking Champions who led the walks. All those who volunteered for this scheme were invited and agreed to participate, except for one who was not available during the study time. In total seven volunteers were interviewed at the beginning of the programme and five at the end (some had left before the end of the programme and new volunteers joined), three were interviewed twice. Of these nine participants, five were women and four were men. All participants were approached by the scheme organiser in the first instance with a general explanation of the research. Subsequent to this all participants were contacted by email or post with a letter inviting them to take part and a participant information sheet with a clear explanation that there was no obligation to participate. All participants responded and gave written informed consent. All interviews were conducted near the beginning of the scheme, in September–October 2014, and at the end of the funding period, in May–June 2015.

Semi-structured interviews were used following a topic guide developed by SH and AJ to ensure that the processes within a process evaluation were explored (Moore et al., 2015). For the stakeholders, questions included the rationale for the scheme as contained in the funding bid; the context for how the scheme was designed; the mechanism for implementation; evaluation plans and barriers and facilitators to implementation. For the volunteers, questions were around training, personal motivations and objectives for volunteering and their perceived role as community Walking Champions. All interviews were conducted by a female doctoral student (SH). Typically interviews took 45 min.

### 2.3 Additional data

Documentary evidence provided by Norwich City Council, including the original bid document, interim reports and the final outcomes report formed part of the data for analysis (Norwich City Council, 2015b).

### 2.4 Data management and analysis

All 33 interviews were digitally recorded and transcribed (by SH). The principles of thematic analysis were used both in the development of the interview framework and in the analysis of both the interview and documentary data with a framework approach used to manage the data (Braun and Clarke, 2006, Gale et al., 2013, Ritchie et al., 2013). This approach enabled continuous cross-checking between the coding and the source of the data. Initially all stakeholder and documentary data was coded as per the methods of a process evaluation: Description (rationale) for the scheme, context, mechanism for implementation; anticipated outcomes (including evaluation plans). Volunteer transcripts were coded for community knowledge, training and motivations (why and how) for joining and sustaining involvement with the scheme. Secondly, using a more inductive approach, the initial themes were further explored and refined from which higher order themes emerged which represent the key findings of this analysis.

Analysis was led by SH as the main researcher and monitored by regular meetings with both AJ and JC throughout the process for cross checking and interpretation of the data. Management of the data was aided using NVivo 10. The study followed the consolidated criteria for reporting qualitative research (Tong et al., 2007).

## 3 Findings

**Fig. 1:**
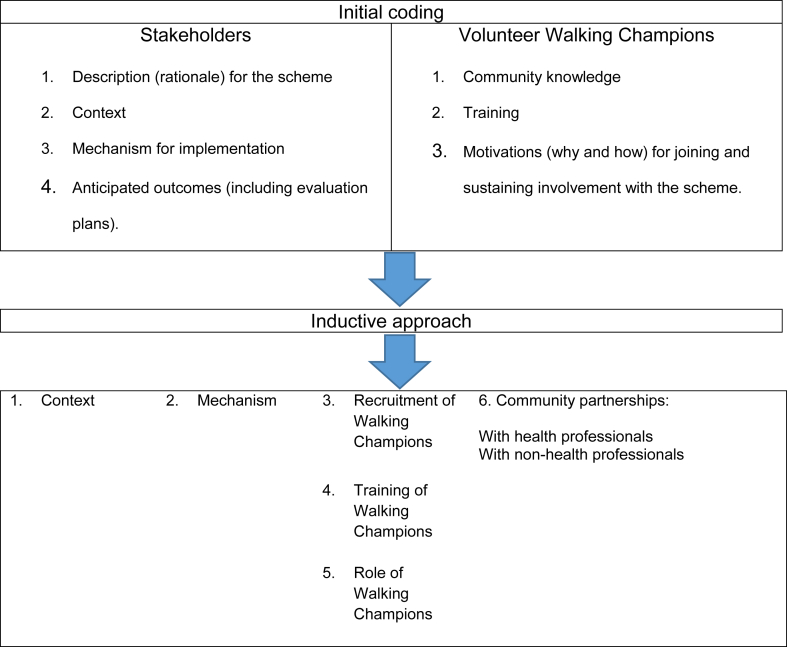
Process and development of main themes.

Data was initially coded around the key functions of a process evaluation for stakeholders and the topics asked of the volunteers. From this, using an inductive approach main themes emerged. This is illustrated in Fig. 1.

The following main themes from the data are supported with illustrative quotes. Stakeholders, volunteers and interview stage is presented as SH, Vol., Int.1 or Int.2.

### 3.1 The context of the programme

The context of the programme primarily came from the documentary data. The programme documents represented this as a 15 month project, funded between the beginning of 2014 to June 2015. £228,500 came directly from the DH and a £12,134 equivalent for supporting the scheme by Norwich City Council. The scheme co-ordinator post cost £96,000 to co-ordinate the three different elements of the project with a £25,000 delivery budget and £37,000 assigned for the health walk element of the programme. In bidding and receiving DH funding, the new scheme aimed to address the health inequalities within Norwich by targeting a new programme of short group health walks at the most inactive. They did this by targeting areas identified through health mapping and local demographic information and professional knowledge.

We looked at not just the physical activity guidelines but the NICE guidelines on walking and looked at the evidence that was out there to support walking and then also at the evidence that we have in the county for stuff that has worked well, or not so well, such as the fit together health walks (Walking for Health scheme). (SH1: Int.1).

### 3.2 Mechanisms for implementing the programme

During the interviews three main themes were identified as mechanisms for the implementation of the scheme. They both facilitated and presented barriers. These are the Walking Champion role; community partnership working and sustaining the scheme beyond the funding period.

### 3.3 Recruitment of the Walking Champions

The recruitment of appropriate Walking Champions was viewed as key to the success of the scheme. Stakeholders were keen that their Walking Champions were representative of the deprived communities they were targeting. For example:

I would like to see them (Walking Champions) recruited from job centres, NEET (not in employment, education or training) young people, people out of work, children out of care, those hard to reach communities and we should recruit from there. We should support them to do the work rather than, yet again, recruiting and investing in professionals. (SH2:Int.2)

I think with the Walking Champions it is really important that it is not just the usual suspects. (SH7: Int.1).

The previous quote appeared to reflect previous findings that membership of walking groups is primarily by professionals who tend to further recruit from the retired, middle classes and women (Matthews et al., 2012). Recruitment of walkers by ‘word of mouth’ was a key recruitment strategy outlined in the bid document and it was envisaged that the Walking Champions would promote the scheme and, *‘spread the word’* to enable the recruitment of walkers into the scheme *(SH5: Int.1).*

The Walking Champions were primarily recruited through newspaper publicity and also via a website (Active Norfolk, 2015). This attracted people local to the area and students in further and higher education. There were differing views on how successful this method was at both recruiting people in the targeted areas and those who would maintain a long term commitment to the scheme.

The range of people we got was exciting. Some local people who have lived here all their lives, students who are in a relevant field and other random locals so it felt really positive. (SH6: Int1).

One of the hardest steps is to get volunteers in those communities. The concern is that they get disheartened because they haven't had the people walking so we need to crack that so we can keep them. (SH8: Int1).

The volunteers also talked about other ways they had been recruited to the scheme.

It was advertised somewhere. I went to the GP [General Practitioner – a family doctor] for an update and there was an A4 brochure about the walks in the waiting area and I thought I'd like to do that. (Vol 8: Int. 2).

Some stakeholders expressed that they would like to have seen a more direct approach by working with the targeted communities to recruit volunteers.

You find champions in the community and you tap into that. (SH3: Int. 1).

Interviews with the volunteers revealed mixed success at recruitment from within the communities that were being targeted. In fact only one of the nine volunteers came from the targeted community, although one had lived there in the past.

Yes, I am from the (targeted community) area and do other volunteering there. (Vol 9: Int. 2).

Where I am doing these walks isn't my neighbourhood, no. It is an area I have known a bit in the past but if I wasn't going there to volunteer I probably wouldn't go there often myself. (Vol 3: Int. 2).

I think it has been good as not coming from this community originally it has given me more knowledge of the community and knowing what's going on and getting out and involved. (Vol 6: Int. 1).

Whilst no longer living in the targeted community, one participant expressed an interesting insight into group walks.

I think if you lived on those estates you wouldn't necessarily want to walk on them where people can see you and you'd rather travel to somewhere else. (Vol 2: Int. 2).

A pragmatic view was also expressed by stakeholders, that whilst the Walk Champions might not have come from within the deprived communities, as intended, volunteers such as university students added useful capacity when the scheme started.

I think our walk leaders are very similar to our walkers, probably 5 or 6 really committed volunteers. The other leaders (students) have added something too, massively, at critical times. (SH 6: Int.2).

There was an expectation in the bid document that the walk leader training would enable the scheme to build sustainability beyond the life of the DH funding. However, there was some reticence expressed about the sustainability of the Walking Champions to have this capability, such as the students leaving the area after graduating.

Are the students going to continue as Champions when they graduate? If they do I would be really chuffed but if they don't it would be wasted. (SH 5: Int.1).

When you have trained someone to be a Walking Champion, how often do they lead a group? How many duties do people do to make use of the knowledge from the training and justify the expense of the training? (SH 5: Int1).

### 3.4 Training of Walking Champions

The bid document stated that Walking Champions would be trained in motivational interviewing and would monitor the progress of participants to the scheme. They would also be offered the Royal Society for Public Health (RSPH) health and wellbeing qualification (Royal Society for Public Health, 2015). In the event, this was different and all volunteers received the less extensive one day ‘Walking for Health’ training to be a walk leader, delivered by a local training co-coordinator (Walking for Health, 2015). This ensures that walks are safe and well run and that walk leaders are ambassadors for walking. However, one stakeholder had a greater expectation of the level of training they would receive.

That Walking Champions are trained as health champions with RSPH, a very basic course but health champions are expected to have that and also some training around behaviour change, very basic psychological stuff, it wouldn't take a lot. (SH2: Int.2).

The volunteers were all positive about their training for their role in leading a group walk.

I thought it was good grounding but again when you are done you are left on your own to progress and it is up to you what you make of it. You get a talk and a folder that outlines the health benefits of walking physically and mentally and how to behave in terms of greeting people and thanking them and inviting them to the next one. (Vol 1: Int.1).

It was the essential stuff, the mechanisms of the scheme, making sure you don't discourage people. (Vol 5: Int. 1).

### 3.5 Role of Walking Champions

Subsequent to the walk leader training, there were differing expectations by the stakeholders of what the Walking Champions were expected to do, beyond leading a walk and completing attendance registers. The following comments at the end of the funding period appear to reflect expectations of a wider remit from the role, more than ‘just’ leading walks, although both had very different expectations of what this was.

I would like to see it being much more holistic and them being able to support on a range of issues and being able to signpost to services and to champion that work and be a motivator in that community … A much more holistic vision of health improvement and supporting people in a local area. It is not just walking. (SH 2: Int. 2).

There is the obvious leading walks, being trained up and being able to set up walks with local people, and leading walks but then there is the other aspect of being the advocate in the neighbourhood in terms of issues relating to streets and a champion for improving the local area in terms of walking … the ideal would be that they built up their skills to know the day to day issues of how their local streets work. (SH 12: Int. 2).

### 3.6 Community partnerships

The scheme aimed to work with GPs, health trainers and community engagement officers in the key deprived areas to ensure the project reached its target audience and to encourage health professionals to refer patients onto the health walks.

### 3.7 Community partnerships with health professionals

Engagement with health professionals remained limited, even at the end of the scheme. The final evaluation showed 10% of walkers had been recruited via booklets in GP surgeries and 31% by word of mouth. In fact finding a booklet in a library (14%) was more popular than a surgery.

We need more referrals from health professionals and health trainers for the short walks that key individuals in surgeries actually get them (walks brochure) and give them to people, otherwise we just drop them off and they go into waste paper. It is key to the short works that they are given by the health professional and that is what is missing. That is the missing link. It always has been. (SH 9: Int.2).

One stakeholder went as far as to say that doctors supporting the benefits of walking would be an achievement in itself.

One of the consolation prizes would be, that success looks like more GPs understand that walking is a great way for patients to improve their health. (SH5: Int.1).

### 3.8 Community partnerships with non-health professionals

The scheme originally aimed to attract walkers by mass publicity with new material, such as brochures. They also expected synergies between the schemes. For example, that the walking to schools project would have cross overs with parents joining the walks after school drop offs. When this did not transpire in the recruitment of participants they changed approach to a community based model, working from community centres with non-health professionals.

The key thing is that where it has been successful it is because of a shared agenda – like St X church … and the parish nurse was a good edition. For ongoing work we would need to refine the community walk hub model as something that we can share and approach with other people of how to set up a community based model. I think we can use the community hubs in the future for more targeted work … you have already got a partner so delivery becomes a lot easier because you don't have to find people. (SH 6: Int.2).

Stakeholders articulated that the scheme had neither located nor utilised those pre-existing assets within the target communities.

I think we try too hard to get people to come to us, rather than going to them and tapping in to existing communities, groups that already get together, rather than constantly re-creating new groups … A really clear audit of what was already happening so that could be built on, where success is already there, build on it rather than try to recreate it. (SH 8: Int.2).

I am amazed at how many organisations already do walks, very small and don't tell anyone about it particularly very much. (SH 6: Int.1).

### 3.9 Sustainability of the scheme

The need to be self-sustaining at the end of the funding period and the issue of securing long term sustainability was raised by stakeholders during both sets of interviews.

We have to engage and empower communities right at the beginning of the project so they feel ownership, they helped to design the project … What we tend to do is write the bid, decide on our project then we engage the community. (SH1. Int.1).

During both sets of interviews, the sustainability of the scheme, funding and long term support was expressed in frustrated terms by stakeholders.

The structure within which we work, financially and politically is inherently short term and yet the benefits are long term … the drivers and incentives are short term but everyone knows that these are long term changes that we want to initiate'. (SH 7: Int.1).

Whilst it was acknowledged that funding for such initiatives had to be replaced by a self-sustaining model, *‘Like all good projects the funding has to stop and at some point it has to self-sustain’ (SH 5: Int.1)* there was much dissatisfaction about what was seen as unrealistic time frames and the management of the funding stream.

People aren't having the chance to invest for a long enough period of time … You can't do community led health improvement over a year or even two years. Our recent evaluation of our healthy community's project was a minimum of 5 years to see real impact. (SH1: Int.2).

The impact on future partnership working with other projects in addition to the effects this has on the community was also voiced.

It is always such short funding and limited and that de-motivates people and prevents engagement. (SH2: Int.1).

There is no scaling up because there is no money or capacity to do it, particularly a scheme that is run by volunteers. To keep volunteers motivated you need to train them and give them reasons to be involved. It will need additional resource but we have the exact opposite when the resource has been withdrawn, so how do you sustain it now? (SH8: Int.2).

There was also a feeling expressed that in order to secure funding the scheme needed to adapt and have a wider offer.

It is only looking at physical activity, it's blinkered and if you are looking for additional funding we would like a broader, wider approach so we would like to see health champions who do walking but can do a whole range. To get funding from us, that would have to be the approach because with the ‘every contact counts’ strategy we really need to see that happen. (SH2: Int.2).

Sustainability in terms of supporting and securing the ongoing commitment of volunteers was also voiced.

Support these people (the Walking Champions), then a year or twos time you have people with all these skills and local experience and they can take on all sorts of new tasks in the local place. (SH12: Int.2).

The problem is as much as you say they will run themselves after you have finished they don't. You always need some sort of paid co-ordinator. (SH1: Int.2).

## 4 Discussion

This paper presents the evaluation of the process of implementing, promoting and sustaining a new group walking scheme in an area of deprivation with poor health indicators. Full outcomes for the scheme can be found electronically, Norwich City Council (2015b). Broadly the scheme provided 185 group walks for 104 new walkers with 691 people attending walks (average 2 per walk with 2 volunteers) from June 2014 until June 2015. Three interrelated factors influenced the intervention's implementation: utilising community based assets, collaborative partnerships with health and non-health organisations and the sustainability of the scheme.

The traditional health care sector, focusing on sickness, finds itself unable to respond to the many determinants of health. Internationally, collaboration and utilising resources within a community is viewed as necessary to promote population health and wellbeing (HM Government, 2010, Hopkins and Rippon, 2015, World Health Organization, 2013, World Health Organization, 2015). To address this nationally, responsibility and accountability for public health in England was devolved from the National Health Service into local government from April 2013. This changes the way that health services are delivered recognising that participatory approaches and empowered communities address the, ‘marginalisation and powerlessness caused by entrenched health inequalities’ (Public Health England, 2015, p. 5). This approach includes the utilisation of community volunteers and the building of collaborations and partnerships; two of the factors found to have influenced the implementation and sustainability of the walking group scheme evaluated here.

The first of these community-based assets is the use of community volunteer Walking Champions. There is recognition that three million volunteers involved in the provision of health and social care is a huge asset to the nation's health (Public Health England, 2015). The role of the ‘expert’ patient includes assisting other patients and was recognised in 2004 in the Wanless report (Wanless, 2004). Such lay health trainers have been effectively used in health behaviour change to improve modifiable lifestyle factors (Barton et al., 2012); in diabetes prevention (Norfolk and Norwich University NHS Foundation Trust, 2015) and as volunteers to assist in walking group programmes (Walking for Health, 2015). Especially important, this approach has shown promise amongst disadvantaged groups. For example, the ‘Altogether better’ programme in Yorkshire and Humberside in England which utilises 17,000 volunteer health champions, working in primary and secondary care to transform health and well-being in their communities (Altogether better, 2015). Additionally, a project in a deprived community in London found that not only was participating in community projects valued by participants but that it also improved social capital and social cohesion (Williams, 2011, p. 11). It is thus seen that utilising community-based assets, such as volunteers in community programmes can improve social capital and individual health and wellbeing in deprived communities (Buck and Gregory, 2013, Hopkins and Rippon, 2015). This evaluation found little evidence that the scheme had recruited Walking Champions that were representative of the deprived communities which were targeted. This may have been due to reliance on media publicity when the scheme was launched and ‘word of mouth’ rather than targeting directly by working with the communities. This is particularly pertinent as part of the Walking Champions role was to be a conduit to recruitment in their own communities.

There was no evidence that the assets needed to achieve change within the community had been identified and mobilised in the planning of the walking group intervention. This is despite evidence that an in-depth understanding of a target group's perspective and involvement in ‘bottom-up’ planning is important in disadvantaged communities (Cleland et al., 2014). Additionally, active recruitment methods (those initiated by the programme) rather than passive (potential participant makes the first contact with the programme), such as ‘word of mouth’ are most effective in engaging hard to reach groups (Matthews et al., 2012). In fact, ‘word of mouth’ is likely to have the potential to increase inequity in walking group membership by utilising social networks that are restricted to the socially well connected. As the scheme moved into a ‘community hub’ model making connections and forming partnerships in the targeted communities, the numbers of walkers increased. These partnerships and new walkers form a pool of potential volunteers to sustain the scheme for the future at the end of the funding period. As has been found in work with peer-support smoking cessation, capacity building is more likely to be effective if people are trained from their own social network within disadvantaged groups (Ford et al., 2013).

There was a mismatch in the expectations of what a Walking Champion might actually do between the different stakeholders which possibly represented a missed opportunity for the Walking Champions to have greater involvement in the scheme's remit. This was in part due to the involvement of two different national charities in the scheme. One was responsible for the initial setting up of the scheme; the training of the Walking Champion and attendance monitoring; the other with day to day management and co-ordination of the other strands of the programme. The agenda for the former is the provision of health walks and the latter campaigns for safe streets for pedestrians (Living Streets, 2015, Walking for Health, 2015). Thus whilst the Walking Champions understood their role as leading health walks, there was an expectation of a much wider remit, such as street audits, signposting to other services and a greater role as a health ambassador. As poor heath behaviours tend to cluster and the responsibility for public health in England has transferred into local authorities there is an increasing expectation for commissioned services to be less ‘siloed’ (Buck and Gregory, 2013, House of Commons Communities and Local Government Committee, 2013). It is possible therefore that those looking to commission health services in the future will look for a wider responsibility for volunteers in championing multiple health behaviours, rather than single interventions.

The second factor that influenced the effectiveness of the implementation of the walking group scheme was collaborative partnerships with health and non-health organisations. There is an expectation in health promotion of community engagement, collaboration and partnership working with local services (Public Health England, 2015). Additionally, physical activity interventions in disadvantaged communities are most effective when there is a mix of professional guidance, self-direction and on-going support (Cleland et al., 2012). Although there was some success in starting to engage with local community groups, engaging health professionals was perceived as the ‘missing link’ that had not been achieved to maximise the impact of the scheme. The group walk was approximately one mile, on an even surface and tailored to those in poor health and inactive. This contrasts with other health walks which tend to be more challenging (Walking for Health, 2015). Therefore targeted referrals to the scheme of people in poor health and inactive by GPs and other health professionals would be most appropriate, and also potentially lead to the greatest gain in public health (de Souto Barreto, 2015).

This evaluation demonstrates the key role that healthcare professionals have in recommending physical activity across the life course. The Health Survey for England reported that whilst only 3% of people would respond to more government advice, 28% would respond to advice to be more active from a doctor or nurse (The NHS Information Centre, 2008). However, despite there being 185 million GP consultations every year, presenting a huge opportunity to promote physical activity, 54% of patients report not being given diet and exercise advice by primary care practitioners (Department of Health (2008)).

The third factor that affected the implementation and impact of the scheme was sustainability. Despite being well funded there were frustrations at the unrealistic timeframe and significant resources spent investigating a means of future funding. This could have been avoided with staged funding over a longer time period. It is noteworthy that at the time of writing this paper, further funding had not been secured to run the scheme and the group walking provision across the county was being re-structured to achieve a more sustainable model. There was also a weariness with short-term interventions done ‘to’ rather than ‘with’ a community. This was despite the acknowledged importance of sustained engagement and better capacity building to leave a positive lasting legacy embedded within a community (Goodman et al., 2014, Hopkins and Rippon, 2015). The ‘hand-to-mouth’ struggle for financial stability may lead to programmes focusing on numbers attending rather than who is being recruited (Matthews et al., 2012). There were concerns that this affected building productive partnership arrangements within a community in the future. This is consistent with recent findings that whilst community interventions can be effective in reducing inequalities in health, there needs to be a greater emphasis on long term outcomes (O'Mara-Eves et al., 2013).

## 5 Strengths and limitations of our study

Strengths of this study is the diversity and number of stakeholders and volunteers who participated. Most were interviewed on two occasions enabling the process of the development of the scheme to be thoroughly evaluated. The scheme organisers were also open to sharing their documentation and all data were analysed using a rigorous theory based thematic analysis. Limitations to this study include that the researcher (SH) was a known volunteer with this and other walking groups. Whilst this appeared to aid rapport and willingness to be interviewed there is a possibility that the research is not seen as neutral, participants may have been more willing to portray the scheme positively and this could have added bias to the findings. The area of this study has a lower ethnic density and mix than many other local authorities in England and future studies would benefit from exploring the experiences of implementing walking groups in more diverse communities.

## 6 Conclusion

**Fig. 2:**
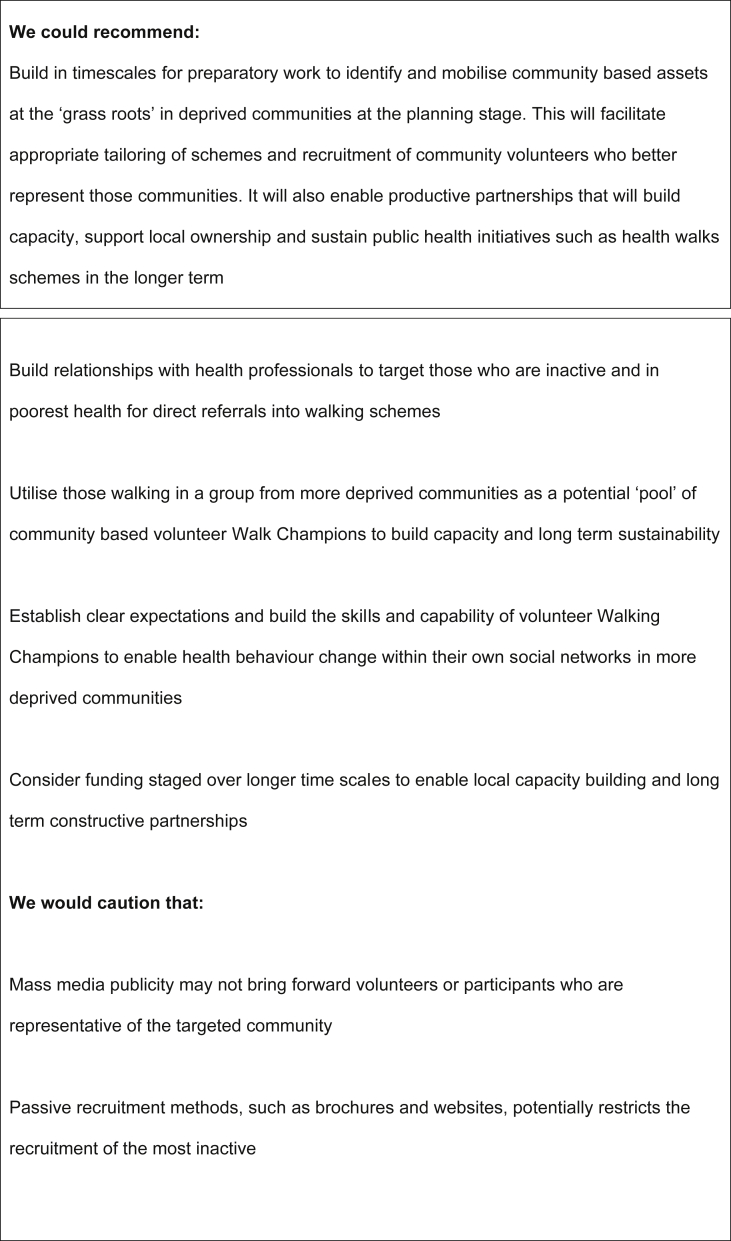
Recommendations to maximise implementation of walking groups in deprived communities.

Whilst walking groups have health benefits concerns exist that they might not operate in areas with the greatest health needs. This study explored factors that facilitated and presented barriers to the implementation and long term sustainability of walking groups in more deprived communities. Our recommendations are summarised in Fig. 2.

It is of concern that ‘yet again’ a public health intervention, with proven efficacy has not been effective when implemented in ‘real world’ circumstances. The evidence that public health initiatives can be successful in deprived communities, and the new supportive structures for community based initiatives that work **with** the assets within communities, represent very real opportunities for ‘grass roots’ public health schemes. We suggest that such initiatives in the future build in a timescale that enables preparatory groundwork with targeted communities to enable interventions to be appropriately tailored. The subsequent use of an asset based partnership model is more likely to result in an appropriate scheme that is owned and sustained after central funding and support has ceased. This may help to stem the flow of initiative fatigue in deprived communities.
